# Identification of alcohol preference relevant genes in QTL on mouse chromosome 2

**DOI:** 10.1186/1471-2105-12-S7-A2

**Published:** 2011-08-05

**Authors:** Lishi Wang, Yan Jiao, Griffin Gibson, Xiaoyun Liu, Yue Huang, Beth Bennett, Kristin Hamre, Robert Williams, Weikuan Gu

**Affiliations:** 1 Department of Orthopedic Surgery - Campbell Clinic and Pathology, University of Tennessee Health Science Center, Memphis, TN, 38163, USA; 2 Department of Pharmacology, University of Colorado Denver, Aurora, CO, 80045-0508, USA; 3 Department of Anatomy and Neurobiology, University of Tennessee Health Science Center, Memphis, TN, 38163, USA

## Background

Previously, a quantitative trait loci (QTL) for alcohol preference on chromosome 2 in a C57BL/6IBG (B6) background has been identified. The overlap of two of interval specific congenic recombinant strains (ISCRS) strains reduced the QTL interval into a 3.4 mbp region.

## Results

By using the keyword alcohol, we identified a total of 39 genetic elements in the region between markers D2Mit56 and D2Mit10. Among these genetic elements, we found seven with potential function in alcohol preference (Table 1). We then examined the SNPs, insertions and deletions, and gene expression levels of those seven genes.

**Table 1:** Candidate genes for alcohol preference on Chr 2.

ENSEMBL ACCESSION	SYMBOL	FULL NAME	SNPS	INSERTIONS	DELETIONS
ENSMUSG00000027104	ATF2	ACTIVATING TRANSCRIPTION FACTOR2	3		
ENSMUSG00000027109	SP3	TRANS-ACTING TRANSCRIPTION FACTOR3	1(G/A)		- AT(72784944)
ENSMUSG00000006494	PDK1	PYRUVATE DEHYDROGENASE KINASE, ISOENZYME 1			-T(71718212)
ENSMUSG00000009207	LNP	LIMB AND NEURAL PATTERNS	9	6	- TA(74365654)
ENSMUSG00000027107	CHRNA1	CHOLINERGIC RECEPTOR, NICOTINIC, ALPHAPOLYPEPTIDE1			
ENSMUSG00000018770	ATP5G3	ATP SYNTHASE, H+ TRANSPORTING, MITOCHONDRIAL F0 COMPLEX, SUBUNIT C, (SUBUNIT 9), ISOFORM 3			
ENSMUSG00000051747	TTN	CONNECTIN	1		

## Conclusions

Our current data suggest that the Atf2 and Titin genes are potentially the most alcohol relevant genes. However, further experiments and examination are still needed to confirm their candidacy. Several other candidate genes are also in the process of being identified.

